# Proteomic profiling reveals insights into Triticeae stigma development and function

**DOI:** 10.1093/jxb/eru350

**Published:** 2014-08-28

**Authors:** Nazila Nazemof, Philippe Couroux, Christof Rampitsch, Tim Xing, Laurian S. Robert

**Affiliations:** ^1^Agriculture and Agri-Food Canada, Eastern Cereal and Oilseed Research Centre, 960 Carling Avenue, Ottawa, ON, CanadaK1A 0C6; ^2^Carleton University, Department of Biology, 1125 Colonel By Drive, Ottawa, ON, CanadaK1S 5B6; ^3^Agriculture and Agri-Food Canada, Cereal Research Centre, 101 Route 100, Morden, MB, CanadaR6M 1Y5

**Keywords:** Electrophoresis, mass spectrometry, proteomics, stigma, triticale, Triticeae.

## Abstract

To our knowledge, this study represents the first high-throughput characterization of a stigma proteome in the Triticeae. A total of 2184 triticale mature stigma proteins were identified using three different gel-based approaches combined with mass spectrometry. The great majority of these proteins are described in a Triticeae stigma for the first time. These results revealed many proteins likely to play important roles in stigma development and pollen–stigma interactions, as well as protection against biotic and abiotic stresses. Quantitative comparison of the triticale stigma transcriptome and proteome showed poor correlation, highlighting the importance of having both types of analysis. This work makes a significant contribution towards the elucidation of the Triticeae stigma proteome and provides novel insights into its role in stigma development and function.

## Summary

This study represents the first characterization of a Triticeae stigma proteome and highlights numerous proteins important in stigma development, pollen–stigma interactions, and protection against biotic and abiotic stresses.

## Introduction

Triticale, a cross between wheat (*Triticum* sp.) and rye (*Secale* sp.), is the first man-made cereal crop. It is more productive than other cereals under poor soil and environmental conditions, less susceptible to many diseases affecting wheat, and competitive with other cereals in terms of grain yield (Chapman *et al.*, 2005). Triticale is also considered a reduced-input crop as it requires less fertilizer and water, and fewer pesticides. Moreover, triticale has a number of potential advantages as a feedstock for bioethanol production (Pejin *et al.*, 2012). Lastly, triticale represents an interesting opportunity to study the implications of allopolyploidization (Ma and Gustafson, 2008).

In flowering plants, pollination is a critical step in sexual reproduction leading to seed production. In triticale, as in other members of the Triticeae, the process begins with adhesion of the pollen grain to the stigma (the receptive portion of the pistil). The pollen grain then rehydrates and germinates, and the newly formed pollen tube penetrates the stigma, grows down the transmitting tissue of the style, and ultimately reaches the ovule, allowing fertilization to take place (Lausser and Dresselhaus, 2010). The stigma plays a critical role in the capture, recognition, and discrimination of the pollen grain, and in supporting pollen germination and tube growth. Generally, it is a relatively short-lived plant structure and has been broadly divided into two major types, wet and dry stigmas (Heslop-Harrison and Shivanna, 1977). In species with a wet stigma, such as in the Solanaceae and Liliaceae, the epidermis is covered with a viscous surface secretion containing protein, lipid, polysaccharides, and pigments, and the stigma has three distinct zones: an epidermis with papillae, a subepidermal secretory zone, and a zone of parenchyma ground tissue (Kandasamy and Kristen, 1987). In species with a dry stigma, such as in the Brassicaceae and Triticeae, the stigma is composed of specialized papillar cells covered with a waxy cuticle overlaid with a distinct proteinaceous pellicle layer, which interacts directly with the pollen (Zinkl *et al.*, 1999; Ciampolini *et al.*, 2001). Compared with the wet stigma, pollen capture, adhesion, and hydration on the dry stigma is a more highly regulated process (Wolters-Arts *et al.*, 1998). The developmental stages of the triticale plumose dry stigma have been described previously (Tran *et al.*, 2013).

In spite of its crucial role, little is known regarding the proteome underlying stigma development and function. High-throughput transcriptome profiling devoted specifically to the stigma has been reported for a few Brassicaceae species (Swanson *et al.*, 2005; Osaka *et al.*, 2013; Sankaranarayanan *et al.*, 2013), rice (Li *et al.*, 2007; Fujita *et al.*, 2010), maize (Davidson *et al.*, 2011; Xu *et al*., 2012, 2013), and triticale (Tran *et al.*, 2013). Only a few studies have investigated the stigma proteome and these have usually focused on a subset. In a comparison between species with wet or dry stigmas using two-dimensional electrophoresis/mass spectrometry, stigma-specific or -preferential proteins were identified from maize silk (67 proteins) and tobacco stigmas (47 proteins) (Sang *et al.*, 2012). These authors also identified 132 proteins present within the tobacco stigma exudate, whereas a separate study revealed 51 and 57 exudate proteins from lily and olive (Rejón *et al.*, 2013). In an analysis of *Brassica* stigmas, differential in-gel electrophoresis/mass spectromtery was used to identify 19 proteins downregulated following self-incompatible pollination, thus bringing new insights into this process (Samuel *et al.*, 2011). Lastly, isobaric tags for relative and absolute quantitation, comparing two developmental stages of *Solanum pennellii* stigma/styles, identified 2534 proteins and highlighted proteome changes associated with stigma/style maturation and the onset of reproductive barriers (Chalivendra *et al.*, 2013).

The current study represents the largest contribution towards a comprehensive survey of the stigma proteome of any Triticeae species. The possible role of the identified triticale proteins in stigma development and pollen–stigma interactions, as well as stress tolerance, is discussed.

## Materials and methods

### Plant material

The hexaploid triticale spring cultivar (×*Triticosecale* AC Alta) was grown as described previously (Tran *et al.*, 2013). In this study, the stigma refers to both the stigma and the style, as these structures are merged in triticale. Mature triticale stigmas were harvested just prior to anthesis (at this stage, the stigma is most receptive to pollen and there is no pollen contamination), frozen immediately in liquid nitrogen, and kept at –80 °C. Bread wheat cultivar Thatcher (*Triticum aestivum* L.) was grown similarly until the first two true leaves emerged.

### Protein extraction

Different procedures were tested to extract triticale stigma proteins, and the most comprehensive and reproducible results were obtained using either SDS-PAGE or isoelectric focusing (IEF) loading buffer directly (results not shown). Frozen stigmas were ground to a fine powder on dry ice and the frozen powder was immediately suspended in 1× SDS-PAGE loading buffer (10% glycerol, 2% SDS, 100mM dithiothreitol (DTT), 1% bromophenol blue and 60mM Tris/HCl, pH 6.8) and sonicated three times for 10 s each using an Ultrasonic Homogenizer 4710 series (Cole-Parmer, Montréal, Canada). After centrifugation at 27 200*g* for 10min at 18 °C, the supernatant was transferred to another tube and heated for 5min at 95 °C prior to one-dimensional (1D) SDS-PAGE. For the IEF loading buffer extraction, the frozen powder was immediately suspended in sample buffer (8M urea, 2M thiourea, 2% CHAPS, 0.1% ampholytes (pI range 3–10), 50mM DTT, 0.0005% bromophenol blue) and incubated at room temperature on a rotator overnight. The sample was then sonicated three times for 10 s each and centrifuged at 48 400*g* for 30min at 4 °C. Total protein concentrations were determined with a Bio-Rad protein assay kit (Bio-Rad, Mississauga, Canada) according to the manufacturer’s instructions.

Wheat leaf proteins were extracted by grinding a 2g aliquot in liquid nitrogen, mixing with 25ml of 50mM Tris/HCl (pH 8.3), 2% (v/v) 2-mercaptoethanol, 20mM MgCl_2_, 1mM PMSF, and grinding further. The sample was centrifuged at 10 000*g* for 10min at 4 °C and the supernatant frozen overnight at –80 °C. To deplete ribulose bisphosphate carboxylase (RbcL and -S), the sample was thawed at room temperature for 2h and then heated to 42 °C with gentle rotation for 15min. This was followed by centrifugation as above at room temperature. Proteins in the supernatant were precipitated with 9 vols of ice-cold acetone containing 0.7% (w/v) DTT and the pellet was dissolved in 1M urea, 50mM Tris/HCl (pH 8.3), 0.7% (w/v) DTT, sonicated, and clarified by centrifugation as above.

### 1D liquid chromatography/tandem mass spectrometry (LC-MS/MS)

Stigma proteins (45 μg) extracted with SDS-PAGE loading buffer were submitted to the McGill University Genome Québec Innovation Centre (http://gqinnovationcenter.com/index.aspx) where the protein sample was loaded on a 2.4cm 1D SDS-PAGE gel with a 7–15% acrylamide gradient. The gel was stained with Coomassie Brilliant Blue G (Supplementary Fig. S1 at *JXB* online) and the entire lane was excised into 15 bands, which were further subdivided into six to seven pieces using a Protein Picking Workstation ProXCISION (PerkinElmer, Shelton, USA). Gel pieces were subjected to reduction, cysteine alkylation, and in-gel tryptic digestion in an automated MassPREP Workstation (Micromass, Manchester, UK) as described previously (Wasiak *et al.*, 2002). A 20 µl volume of tryptic digest solution was injected into a Zorbax 300SB-C18 column (Agilent Technologies, Mississauga, Canada) at 15 µl min^–1^, and the sample was washed for 5min and then analysed with a Q-Tof micro^TM^ MS (Waters Corporation, Milford, MA, USA). The MS survey scan was set to 1 s recording from 350 to 1600 *m*/*z* with all doubly and triply charged ions with an intensity higher than 25 counts considered candidates to undergo MS/MS fragmentation, and from these the strongest one was selected. The MS/MS scan was acquired twice, with the second round excluding the most intense precursor of the first round. The precursor was excluded within ±1900 mDa of the entries on the exclusion list.

### Two-dimensional (2D) LC-MS/MS

**Fig. 1.**
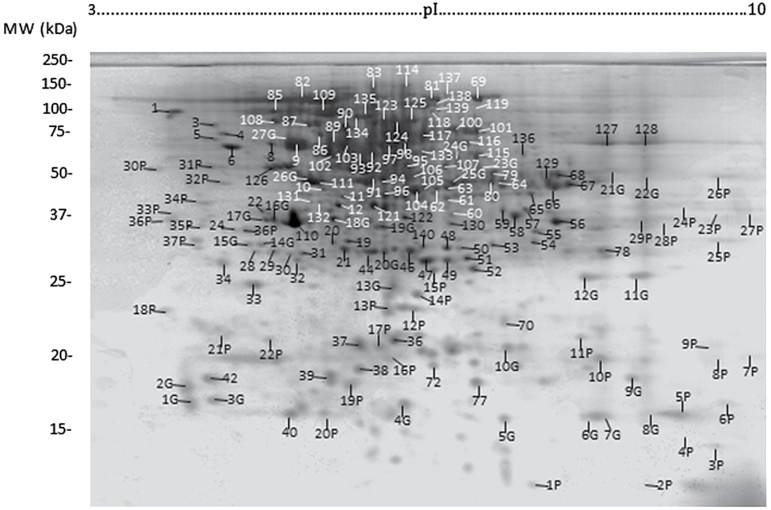
2D gel electrophoresis of triticale mature stigma proteins also indicating the most highly stained proteins observed in a separate experiment investigating post-translational modifications. P, Pro-Q Diamond phosphoprotein stain; G, Pro-Q Emerald glycoprotein stain (Life Technologies, Burlington, Canada).

For 2D gels, stigma proteins extracted with IEF loading buffer (200 µg) were first separated on a 17cm pH 3–10 gradient using a PROTEAN IEF system (Bio-Rad) according to the manufacturer’s instructions. After IEF, the strips were first equilibrated for 20min in 6M urea, 2% DTT, 375mM Tris/HCl (pH 8.8), and then for another 20min in the same solution containing 135mM iodoacetamide instead of 2% DTT. SDS-PAGE was performed in a 12% acrylamide gel using a PROTEAN II XI Cell system (Bio-Rad) at a constant voltage of 77V. The gels were stained with Coomassie Brilliant Blue R-250 and gel images were captured with a scanner Astra 2400 SLT (UMAX, Dallas, USA) or Gel Doc XR+ (Bio-Rad). Three 2D gel replicates gave consistent results (Fig. 1). For future reference, the identity of the major triticale stigma proteins was investigated and a total of 170 individual protein spots were excised manually from Coomassie Brilliant Blue stained gels and submitted to the Québec Genomics Center (http://proteomique.crchul.ulaval.ca/en/index.html) for MS. Briefly, the excised proteins were automatically destained, dehydrated, reduced with 10mM DTT, alkylated with 55mM iodoacetamide, and digested with 105mM modified porcine trypsin at 58 °C for 1h using a MassPrep liquid handling robot (Waters Corporation). Digestion products were extracted using 1% formic acid, 2% acetonitrile, followed by 1% formic acid, 50% acetonitrile, and the recovered extracts were pooled, lyophilized, and then resuspended in 7 µl of 0.1% formic acid. A peptide sample volume of 2 µl was separated by online reversed-phase high-pressure liquid chromatography (RP-HPLC) using a Jupiter 300 C18 column (Phenomenex, Torrance, USA) and analysed by electrospray ionization MS/MS. The experiments were performed with a Thermo Surveyor MS pump connected to a LTQ Linear Ion Trap Mass Spectrometer equipped with a nanoelectrospray ion source (ThermoFisher Scientific, San Jose, USA). Mass spectra were acquired using a data-dependent acquisition mode using the Xcalibur software version 2.0. Each full scan mass spectrum (400–2000 *m*/*z*) was followed by collision-induced dissociation of the seven most intense ions. The dynamic exclusion (30 s exclusion) function was enabled and the relative collisional fragmentation energy was set at 35%. As many spots contained more than one protein, a quantitative analysis of relative spot intensity was not performed.

### OFFGEL electrophoresis (OGE) LC-MS/MS

Triticale stigma proteins (90 μg) extracted with IEF loading buffer were sent to Bioproximity Proteomics Services (http://www.bioproximity.com). Briefly, the protein sample was processed using a filter-assisted sample preparation method (Wiśniewski *et al.*, 2009), digested at a 1:40 trypsin:substrate ratio and incubated overnight at 37 °C. Digested peptides were desalted using C18 stop-and-go extraction tips (Rappsilber *et al.*, 2007) and eluted with 60% acetonitrile, 0.5% acetic acid, and lyophilized. Peptides were then fractionated using an Agilent OFFGEL fractionator (Agilent Technologies) with a 24cm pH 3–10 IPG strip in conjunction with a 24-well tray according to the manufacturer’s instructions. Each fraction was desalted as above and analysed by LC-MS/MS. LC was performed using an Easy Nano-LC II HPLC (ThermoFisher Scientific) using a Zorbax 300SB-C18 column (Agilent Technologies). The LC was interfaced to a LTQ Velos Dual-Pressure Linear Ion Trap mass spectrometer (ThermoFisher Scientific) via nano-electrospray ionization. The mass spectrometer was programmed to acquire, by data-dependent acquisition, tandem mass spectra from the top 15 ions in the full scan from 400 to 1400 *m*/*z*. Dynamic exclusion was set to 30 s.

### LC-MS/MS of wheat leaf proteins

To prepare the sample for off-line separation by RP-HPLC, each leaf peptide sample replicate was first desalted using a 5cm RP column (MOS1-Hypersil: ThermoFisher Scientific) connected to an analytical HPLC pump (Ultimate 3000; Dionex, Bremen, Germany). The eluent was monitored at 220nm, and a single desalted fraction was collected. The desalted peptides were separated into 60 fractions using a high-pH acetonitrile gradient as described elsewhere (Dwivedi *et al.*, 2008). Fractions were pooled by interlacing fractions 1+21+41; 2+22+42, etc., resulting in 20 fractions analysed by LC-MS/MS on a linear ion trap mass spectrometer (LTQ XL; ThermoFisher Scientific) as described previously (Rampitsch *et al.*, 2013).

### Data analysis

All MS/MS spectra were analysed using Mascot version 2. 3. 0 (http://www.matrixscience.com) and X! Tandem version 2007.01.01.1 (http://www.thegpm.org/tandem/) to search the Universal Protein Resource (UniProt) Viridiplantae database. The search was restricted to a maximum of one missed (trypsin) cleavage, fixed carbamidomethyl alkylation of cysteine, and variable oxidation of methionine, as well as a 0.5Da mass unit tolerance for fragment ions and 2.0Da for parent ions. The maximum false discovery rate was set to 0.3% for proteins and 5.3% for peptides. Scaffold version 4.0.4 (Proteome Software, Portland, OR, USA) was used to validate MS/MS-based peptide and protein identifications. Peptide identifications were accepted if they could be established at a greater than 95% probability as specified by the peptide Prophet algorithm (Keller *et al.*, 2002). Protein identifications were accepted if they could be established at a greater than 95% probability and contained at least two identified peptides. Scaffold grouped proteins displaying shared peptides to satisfy the laws of parsimony. Proteins with a single peptide match at a greater than 95% probability (Supplementary Table S1 at *JXB* online) were validated manually based on the following criteria: the peptide had to be identified at least by two search engines (Mascot with significant ion score of 25 and X! Tandem), contain a minimum of 80% coverage of theoretical y or b ions (at least three amino acids in a row in either y or b ions series), and show an absence of prominent unassigned peaks greater than 5% of the maximum intensity (Feng *et al.*, 2009; Prenni *et al.*, 2012). SignalP4.1 (http://www.cbs.dtu.dk/services/SignalP) was utilized to predict the presence of signal peptides. Detailed information about specific proteins (e.g. Mascot scores, sequence of peptides identified) can be provided upon request.

To compare triticale mature stigma protein and mRNA data, the 542 protein sequences identified in the 1D SDS-PAGE shotgun analysis were submitted to a tBLASTn search against an in-house triticale reference transcriptome assembly and the 239 contigs whose derived protein sequences showed ≥95% identity were selected for further analysis. These contigs were then aligned to the sequences used to generate the different probe sets of the 55K Affymetrix GeneChip® wheat genome array and the 180 contigs showing ≥95% identity were selected for expression comparisons with the matching proteins. The mature triticale stigma gene expression levels for these 180 contigs were derived from the microarray study (Tran *et al.*, 2013), whereas the normalization of the corresponding spectral counts was performed using Scaffold.

## Results and discussion

### Comparison of 1D LC-MS/MS, 2D LC-MS/MS, and OGE LC-MS/MS

**Fig. 2.**
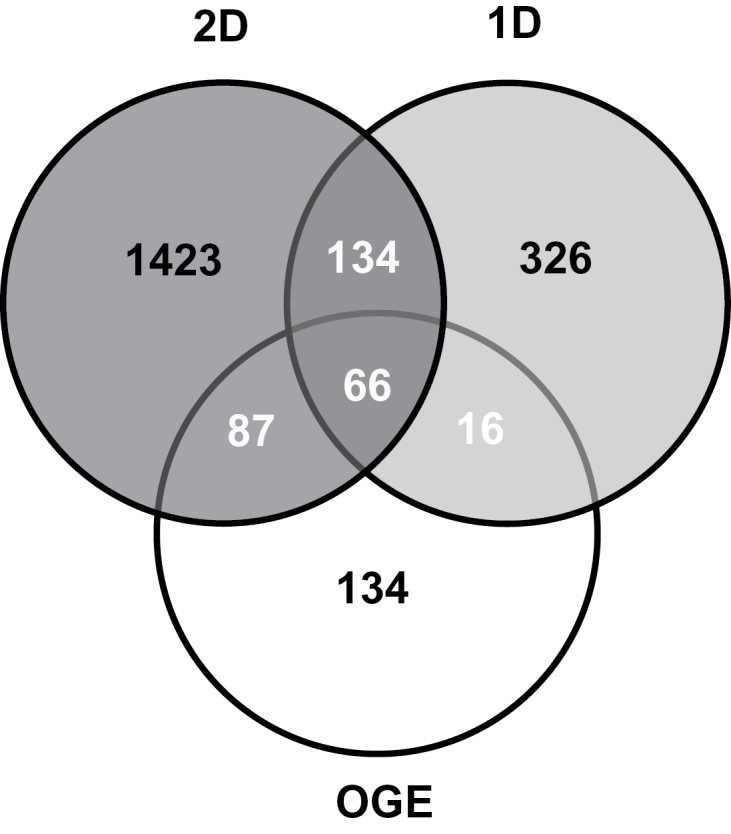
Overlap among the proteins identified by the different methods of analysis. 2D, 2D LC-MS/MS; 1D, 1D LC-MS/MS; OGE, OGE LC-MS/MS.

In this study, the proteome of the mature triticale stigma was analysed using three different electrophoretic approaches. A total of 2555 proteins were identified comprising 542 proteins using 1D LC-MS/MS, 303 proteins by OGE LC-MS/MS, and 1710 proteins with 2D LC-MS/MS, which represented a total of 2184 unique proteins (Supplementary Table S1). We examined the overlap in the proteins identified using the three different proteomics techniques and, surprisingly, only 66 proteins of the 2184 proteins identified in this study were identified by all three approaches and an additional 237 proteins were detected by two of the three methods (Fig. 2). Half of the proteins found with OGE LC-MS/MS were also identified by 2D LC-MS/MS and this may reflect the fact that they share the same protein extraction buffer, although in the 2D LC-MS/MS it was the proteins that were separated by IEF, whereas it was tryptic peptides with OGE LC-MS/MS. These results clearly indicate that different proteomic approaches can yield distinct proteins from the same tissue, and combining different techniques increases the number of identified proteins.

Evidently, the ‘shotgun’ approaches of 1D LC-MS/MS and OGE LC-MS/MS did not uncover as many stigma proteins as 2D LC-MS/MS, and this was probably the result of a lower number of more highly complex samples. Although labour intensive, 2D LC-MS/MS does provide considerable information pertaining to the proteins detected, as well as a protein profile (Fig. 1) that can be used in comparative analyses such as differential in-gel electrophoresis. For example, almost one-third of the 1710 identified proteins were found in multiple spots with different pIs and molecular masses (Supplementary Table S1), pointing to the existence of isoforms possibly resulting from post-translational modifications, multimeric forms of the protein, proteolytic processing, and alternative transcripts. This is a higher proportion than reported for *Arabidopsis* floral buds (Feng *et al.*, 2009) and may indicate a higher degree of post-translational modification within the stigma. The 2D gels were also analysed for the presence of phosphorylated or glycosylated proteins using commercially available stains, but MS analysis of intensely fluorescent spots (labelled P and G in Fig. 1) could not confirm the presence of these post-translational modifications (results not shown).

### Functional classification of triticale mature stigma proteins

**Fig. 3.**
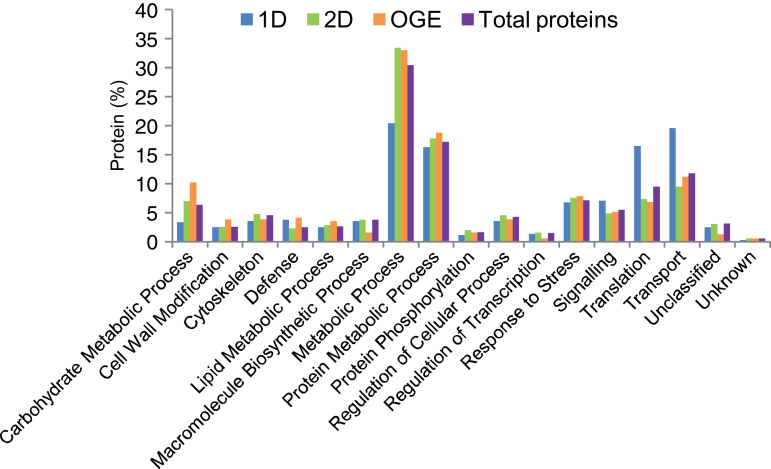
Functional distribution of triticale mature stigma proteins identified using different methods of analysis and their combined total. 2D, 2D LC-MS/MS; 1D, 1D LC-MS/MS; OGE, OGE LC-MS/MS.

Generally, the proteins identified by the different electrophoretic procedures displayed similar overall functional distribution patterns (Fig. 3), although, for reasons that are not evident, 1D LC-MS/MS differed considerably in the categories Metabolic Process, Translation, and Transport. More than 75% of the 2184 triticale mature stigma proteins identified in this study were found in only five categories: Metabolic Process (30.5%), Protein Metabolic Process (17.2%), Transport (11.9%), Translation (9.6%), and Response to Stress (7.3%). A detailed description of the constituent proteins of each functional category is beyond the scope of this article, and the discussion will therefore be restricted to some examples relevant to three critical aspects of stigma biology: stigma development, pollen–stigma interactions, and stigma responses to biotic and abiotic stress.

### Proteins involved in stigma development

Very little is known regarding the identity and function of the proteins underlying stigma development in the Triticeae; however, it is possible to infer a potential role for the triticale stigma proteins identified in this study (Supplementary Table S1) based on homology to proteins shown to play a role in development, primarily in model species. Transcription factors (TFs) are evident candidates as potential regulators of stigma development, and some of the TFs found within the triticale mature stigma included orthologues to TFs known to have a role in gyneocium development including DROOPING LEAF (DL)/CRABS CLAW (CRC), which is involved in determining carpel identity and mid-rib formation (Li *et al.*, 2011), GT2, KNOTTED-LIKE, ARABIDOPSIS THALIANA3 (KNAT3), which, although shown to be involved in embryo sac development in *Arabidopsis* (Pagnussat *et al.*, 2007), was also found in the triticale stigma as a transcript (Tran *et al.*, 2013), and SHORT INTERNODES (SHI)/STYLISH (STY), which regulates auxin-mediated stigma and style development by interacting with YUCCA4 (YUC4) (Larsson *et al.*, 2013). Auxin has been proposed to play a role in stigma and style development and function (Ståldal and Sundberg, 2009), and microarray analysis has shown that auxin-signalling components are over-represented in the rice stigma (Li *et al.*, 2007) and that several auxin response factors show enriched expression in the triticale stigma (Tran *et al.*, 2013). A role for auxin in triticale stigma development is further supported by the presence of orthologues to the *Arabidopsis* proteins HEMIVENATA (HVE), a CULLIN-ASSOCIATED-NEDD8-DISSOCIATED1 (CAND1) protein that negatively regulates ubiquitin-mediated auxin signalling and plays a role in leaf venation and cell division (Pérez-Pérez *et al.*, 2011), and AUXIN RESISTANT1 (AXR1), a RUB/NEDD8-activating enzyme E1 regulatory subunit that plays a role in auxin signalling and in development (Nakasone *et al.*, 2012), as well as the ABC transporter ABCB15 shown to transport auxin (Kaneda *et al.*, 2011).

A variety of additional proteins were identified that may also function in triticale stigma development and these comprised several CELL DIVISION CONTROL (CDC) 48-like proteins, which are transitional endoplasmic reticulum ATPases involved in protein turnover and shown in *Arabidopsis* to affect cell division, expansion, and differentiation, as well as pollen-tube growth (Park *et al.*, 2008). Other triticale stigma proteins with homologues shown to have developmental roles included FLORAL ORGAN REGULATOR-LIKE1 (FORL1) and FORL2, which are related to OsFOR1 involved in regulating floral organ number in rice (Jang *et al.*, 2003), TASSELSEED2 (TS2), which when absent leads to the feminization of maize tassels (DeLong *et al.*, 1993), POINTED FIRST LEAF (PFL), which represents ribosomal proteins that can affect growth and development (Ito *et al.*, 2000), a RAN GTPase-activating protein (RanGAP1) with a conserved function in cell division and important to female gametophyte development in *Arabidopsis* (Rodrigo-Peiris *et al.*, 2011), ABNORMAL INFLORESCENCE MERISTEM1 (AIM1), a fatty acid β-oxidation multifunctional protein (Richmond and Bleecker, 1999), and PEPPER (PEP), a protein containing KH RNA-binding domains that regulates gynoecium development including that of the stigma (Ripoll *et al.*, 2006).

A number of proteins highlight the importance of epigenetic mechanisms in triticale stigma development such as orthologues to the *Arabidopsis* METHYL-CPG-BINDING DOMAIN PROTEIN11 (MBD11), which when downregulated causes developmental defects including abnormal flower positions, delayed flowering, and reduced fertility (Berg *et al.*, 2003), and MULTI-SUBUNIT SUPPRESSOR OF IRA1 (MSI1) involved in gene silencing during female gametogenesis and early seed development (Hennig and Derkacheva, 2009), as well as GLABRA2 EXPRESSION MODULATOR (GEM), which, via histone modification, inhibits the expression of the transcription factor GLABRA2 (GL2) implicated in epidermal cell division and cell fate (Qing and Aoyama, 2012) and which may play a role in Triticeae stigma papillae formation. Lastly, the conservation of mechanisms underlying monocot floral patterning is indicated by the presence of an orthologue to the *Arabidopsis* HISTONE DEACETYLASE19 (HDA19) shown to be part of a repressor complex with APETAL2 (AP2) and TOPLESS (TPL) regulating floral organ identity genes (Krogan *et al.*, 2012).

A role for protein phosphorylation in stigma development is supported by the presence of *Arabidopsis* orthologues CYCLIN-DEPENDENT KINASE F;1 (CDKF;1), a major regulator of cell proliferation and expansion (Takatsuka *et al.*, 2009), different SNF1-related protein kinase (SNRK) regulatory subunits, which can interact with NAC-domain TFs to positively regulate development (Kleinow *et al.*, 2009), CASEIN KINASE2 (CK2) implicated in multiple developmental pathways (Mulekar and Huq, 2014), and 2A PHOSPHATASE ASSOCIATED PROTEIN OF 46KD (TAP46) with roles in development via the TARGET OF RAPAMYCIN (TOR) and ABI5 regulatory pathways (Hu *et al.*, 2014). At present, the great majority of the many protein phosphatases identified in this study could not be assigned a putative role.

The Triticeae stigma is a non-green tissue enclosed within the floret and hence it will be interesting to investigate the role of a number *Arabidopsis* orthologues associated with photomorphogenesis including different subunits of the CONSTITUTIVE MORPHOGENESIS9 (COP9) SIGNALOSOME (CSN), which although involved in photomorphogenesis also regulates many different cellular processes by regulating the activity of CULLIN-RING E3 ubiquitin ligases (Stratmann and Gusmaroli, 2011), TF Z-BOX BINDING FACTOR3 (ZBF3)/CALMODULIN7 (CAM7) that integrates light signals with seedling development (Gangappa *et al.*, 2013), and THYLAKOID FORMATION1 (THF1) whose rice orthologue NYC4 was recently shown to play a role in chlorophyll–protein complex degradation during leaf senescence (Yamatani *et al.*, 2013), as well as SCHLEPPERLESS (SLP)/CHAPERONIN60 (CPN60) and chaperones part of the chloroplast multi-subunit Clp protease such as DE-REGULATED CAO ACCUMULATION1 (DCA1)/ClpC1 and ALBINO OR PALE GREEN6 (APG6)/ClpB3, which are all involved in chloroplast development (Apuya *et al.*, 2001; Sjögren *et al.*, 2004; Myouga *et al.*, 2006).

These few examples illustrate (mostly for the first time) the occurrence of proteins involved in the regulation of Triticeae stigma development and other such examples can be found in Supplementary Table S1.

### Proteins involved in pollen–stigma interactions

The landing of a compatible pollen grain onto the stigma surface marks the beginning of pollen–pistil interactions enabling pollen recognition, adhesion, germination, tube growth, and finally fertilization. Little is known of the molecules involved in the recognition process within the grasses (Klaas *et al.*, 2011). The first layers of contact are the protein pellicle and then the cuticle covering the dry stigma, both of which the pollen tube must breach. The triticale mature stigma expressed many proteases (Supplementary Table S1), and some may facilitate penetration of the protein pellicle. A cutinase responsible for the breakdown of the stigma cuticle has not been identified, but a GDSL lipase was recently shown to fit the description (Takahashi *et al.*, 2010) and one of the GDSL lipases expressed in the triticale stigma could have a similar role. A significant number of other triticale stigma proteins within the Lipid Metabolic Process category are likely to be involved in stigma cuticle formation, and these include orthologues to the *Arabidopsis* FIDDLEHEAD (FDH), which has been shown to play a role in pollen–stigma interactions (Lolle and Cheung, 1993), DEFECTIVE IN CUTICULAR RIDGES (DCR), a diacylglycerol acyltransferase involved in cuticle formation, epidermal cell differentiation, and the regulation of water flow (Panikashvili *et al.*, 2009), a GDSL lipase shown to function in cutin biosynthesis (Shi *et al.*, 2011), an ABC transporter similar to the barley EIBI1, and the *Arabidopsis* ATP BINDING CASSETTEG32 (ABCG32) likely to export cutin precursors from the epidermal cells (Bessire *et al.*, 2011), ECERIFERUM8 (CER8)/LONG-CHAIN ACYL-COA SYNTHETASE1 (LACS1) proteins involved in wax and cutin synthesis (Lü *et al.*, 2009), and ANTHOCYANINLESS2 (ANL2), an HD-START TF that plays a role in epidermal cell development as well as anthocyanin and cutin accumulation (Wu *et al.*, 2011). HD-START TFs represented a major category of TFs enriched within the stigma transcriptome (Tran *et al.*, 2013). Finally, several non-specific lipid transfer proteins were found within the stigma proteome. Lipid transfer proteins have been ascribed a number of different roles in development, signalling, and biotic and abiotic stresses, as well as cutin biosynthesis (Edstam *et al.*, 2011). As with many of the proteins described below, it will be interesting to investigate whether many of these proteins play a role primarily in maturing stigma development/growth or pollen germination/tube growth, or are involved in both activities.

The pollen tube penetrates the stigma cell wall and grows within it, thus necessitating proteins for both cell growth and cell wall degradation and synthesis. The triticale stigma proteome exhibited numerous cell wall-modifying proteins including, for example, many glycosyl hydrolases and several expansins, as well as pectin-modifying enzymes like pectinesterases and pectate lyase (Supplementary Table S1). Other proteins of interest included lipoxygenases, which are known to affect papillae expansion (Caldelari *et al.*, 2011), TASSELSEED1 (TS1), a lipoxygenase necessary for maize pistil primordial abortion possibly via the jasmonic acid pathway (Acosta *et al.*, 2009), dynamin-related proteins, which have a role in cytokinesis and cell expansion including cell wall formation (Collings *et al.*, 2008) and were shown to be involved in *Arabidopsis* stigma papillae expansion (Kang *et al.*, 2003), two CELLULOSE SYNTHASE-INTERACTING (CSI) proteins shown in *Arabidopsis* to bridge cellulose synthase to cortical microtubules, a process underlying directional growth (Li *et al.*, 2012), and multiple versions of UDP-ARABINOPYRANOSE MUTASE (UAM)/REVERSIBLY GLYCOSYLATED POLYPEPTIDE (RGP), which generates UDP-arabinofuranosyl associated with arabinoxylan, an important component of grass primary and secondary cell walls (Konishi *et al.*, 2011). In addition, four different fasciclin-like arabinogalactan proteins were identified; arabinogalactan proteins are known to be abundant in transmitting tissue and are a major constituent of lily stigma exudate (Rejón *et al.*, 2013), while the fasciclin domain involved in protein–protein interactions was shown to be involved in cell adhesion and could also mediate extracellular interactions (Ellis *et al.*, 2010). Lastly, orthologues to the *Arabidopsis* FERONIA (FER) and HERCULES (HERK) were identified within the triticale stigma proteome, and these receptor-like protein kinases are important to cell wall metabolism and cell growth (Lindner *et al.*, 2012).

The growth of the pollen tube through the stigma eventually becomes auxotrophic and relies on support from the surrounding tissue, and this in turn signifies a requirement for the transport of various nutrients and signals. Accordingly, transport is the third largest functional category of the triticale stigma proteins (Fig. 3), consistent with the enrichment of transport-related transcripts in the mature stigma (Xu *et al.*, 2012; Tran *et al.*, 2013). A role for vesicle-mediated secretion of stigma resources has been suggested (Samuel *et al.*, 2011), and a high proportion of the transport proteins of the triticale stigma proteome belong to relevant proteins such as annexins, coatomer subunits, and SEC proteins (Supplementary Table S1). GTPase-mediated regulation of membrane trafficking is also reflected by the high number of associated proteins detected in the triticale stigma proteome including ADP-RIBOSYLATION FACTORS (ARFs), GDP DISSOCIATION INHIBITORS (GDIs), ARF GTPASE-ACTIVATING PROTEINS (ARFGAPs), and PRENYLATED RAB ACCEPTORS (PRAs), as well as many GTPases, most of which are either dynamin-related or Rab GTPases, with the most highly represented GTPase being SECRETION-ASSOCIATED RAS1 (SAR1). The role of Rab GTPases in membrane trafficking and signalling has recently been reviewed (Rehman and Di Sansebastiano, 2014), and characterizing the role of this process in cereal pollen–pistil interactions will be a significant future challenge. The next largest group of proteins in the Transport category was ATPases mainly V-type and P-type proton ATPases important in the creation of gradients underlying membrane potential and ion transport during pollen-tube growth (Hepler *et al.*, 2013), as well as AAA+ ATPases, which are essentially molecular chaperones with a diversity of functions revolving around processes such as protein unfolding or disassembly of protein complexes (Ogura and Wilkinson, 2001).

Stigmas can accumulate high amounts of reactive oxygen species, as well as H_2_O_2_ (McInnis *et al.*, 2006), and these are known to function as signalling molecules in a wide range of processes in plants including stress responses (Foyer and Noctor, 2013). Furthermore, the role of redox regulation in pollen–stigma interactions is becoming increasingly evident (Dresselhaus and Franklin-Tong, 2013; Traverso *et al.*, 2013). The Metabolic Process category contained a high proportion of triticale stigma proteins associated with redox processes (Supplementary Table S1), with examples including many enzymes involved in glutathione metabolism such as glutathione *S*-transferases, peroxidases, reductases, and synthetases, as well as numerous redoxins and enzymes such as superoxide dismutases, ascorbate peroxidases, catalases, peroxidases, malate dehydrogenases, malic enzymes, 6-phosphogluconate dehydrogenases, and isocitrate dehydrogenases. The role of these proteins in Triticeae pollen–stigma interactions remains to be elucidated.

The continuous interaction between the pollen grain/tube and the stigma implies a high degree of communication, and numerous additional triticale stigma proteins likely to play a significant role in signalling were identified including several 14-3-3 proteins, which regulate an ever-growing number of processes such as cell growth and responses to hormones, light, and stress via phosphorylation-dependent associations (Denison *et al.*, 2011), as well as proteins linked to calcium signalling such as calmodulins, calreticulins, CaLB domain-containing proteins, calcium-dependent protein kinases (CDPKs) and several phospholipase D (PLDs), which have been shown to be involved for example in cytoskeletal dynamics, membrane remodelling, and biotic and abiotic stress responses (Wang *et al.*, 2014). The triticale stigma also expressed RECEPTOR FOR ACTIVATED C KINASE 1A (RACK1A) proteins, which are scaffolding proteins that interact with signalling proteins in a variety of signalling pathways associated with development, hormonal, and stress responses (Dongping *et al.*, 2013), and several inositol-3-phosphate synthases responsible for the first step of inositol biosynthesis, a key substrate in the phosphoinositide signalling pathway affecting processes such as growth and development, as well as stress responses (Boss and Im, 2012).

As exemplified above, the interaction between the pollen grain/tube and the stigma operates at multiple levels, and elucidation of the cellular processes involved will be of great interest.

### Proteins involved in biotic and abiotic stress

The stigma and style are meant to be breached by, and provide nutrients to, the growing pollen tube while preventing this from happening with pathogens and this represents a remarkable challenge to the plant. Furthermore, the next generation must also be preserved against abiotic stresses. Accordingly, these tissues often express relatively high numbers of stress-related genes and indeed it has been found that considerable functional overlap exists between reproductive and stress-responsive genes (Li *et al.*, 2007). In this study, this is supported by the fact that many of the proteins involved in stigma development and pollen–stigma interactions discussed above such as AIM1, HDA19, FORL, SNRK, CK2, THF1, APG6, COP9, and PLDs, as well as many of the redox proteins, are known to also play a role in the stress response. The triticale mature stigma expressed a number of proteins with homology to protein kinases known to be involved in the defence and stress responses including ZmCPK11 (Ding *et al.*, 2013), PROTEIN KINASE INTERACTOR1 (PTI1) (Forzani *et al.*, 2011), OsCDPK7 (Saijo *et al.*, 2000), STRESS-ACTIVATED PROTEIN KINASE7 (SAPK7) (Kobayashi *et al.*, 2004), and AtMPK1 (Hwa and Yang, 2008). The TF AtNF-YC2, which is activated by oxidative stress and is involved in flowering (Hackenberg *et al.*, 2012), was also identified in the triticale stigma proteome.

The major class of stress-associated proteins found in the triticale stigma consisted of heat-shock-related proteins represented mainly by HEAT SHOCK COGNATE70 (HSC70), HEAT SHOCK PROTEIN70 (HSP70), and HSP90 proteins (Supplementary Table S1). In fact, nearly half of the Protein Metabolic Process category was made up of proteins involved in chaperone activities also including many PROTEIN DISULFIDE ISOMERASES, PEPTIDYL PROLYL ISOMERASES, and CHAPERONIN60 (CPN60)/T-COMPLEX PROTEIN1 (TCP1) family proteins, all of which may play a role in the stress response. The next largest group of the Protein Metabolic Process category was made up of proteins associated with the proteasome, which not only mediates many developmental processes but also plays a major role in plant responses to stress (Lyzenga and Stone, 2012). One possible example is the triticale orthologue to the *Arabidopsis* TF ABA BINDING FACTOR3 (ABF3), which, like many other proteins in this study, implicates abscisic acid in stress tolerance and was recently shown to be regulated via proteasome-mediated proteolysis (Chen *et al.*, 2013). Stress-related proteins, which were found repeatedly, also included the UNIVERSAL STRESS PROTEIN (USP) family proteins and UVB-RESISTANCE8 (UVR8) proteins (Supplementary Table S1).

Lastly, defence proteins, which appeared often within the triticale mature stigma proteome, included HYPERSENSITIVE-INDUCED REACTION (HIR) proteins, PATHOGENESIS-RELATED (PR) proteins, thaumatin-like proteins, chitinases, ricin B-like proteins, allene oxide oxidases, as well as HEAT repeat-containing ILITYHIA (ILA)-like proteins required for disease resistance in *Arabidopsis* (Monaghan and Li, 2010) and ARGONAUTE4 (AGO4) proteins involved in RNA-directed DNA methylation shown to be involved in bacterial and viral disease resistance (Seo *et al.*, 2013).

Therefore, this study not only confirmed the presence of a high proportion of proteins involved in both biotic and abiotic stress in the stigma, but also showed the occurrence of a considerable functional overlap in many stress and developmental proteins.

### Comparison of the stigma and leaf proteome

**Fig. 4.**
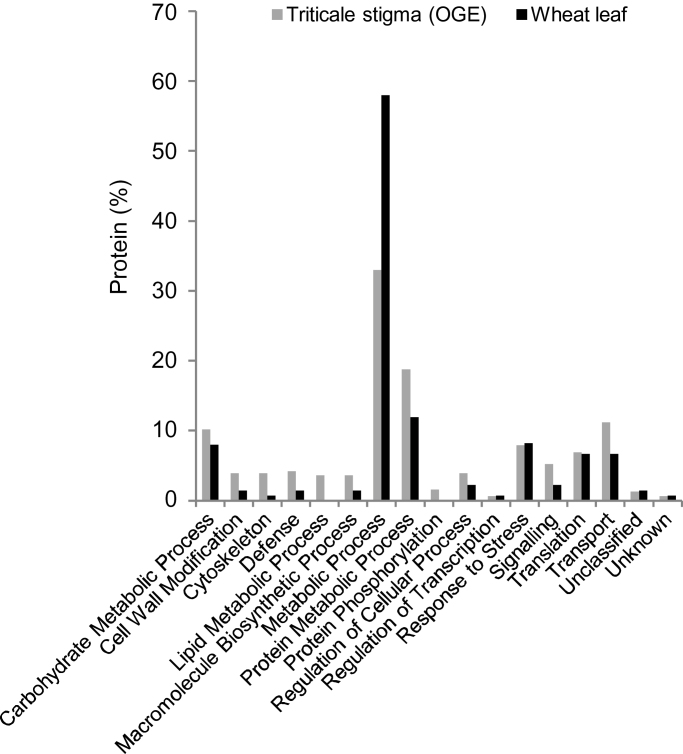
Comparison of the functional distribution of triticale mature stigma and wheat leaf proteins. OGE, OGE LC-MS/MS.

In the hope of highlighting functional categories that may be under- or over-represented within the stigma, the functional distribution of mature stigma proteins (OGE) was compared with that of the wheat leaf proteome obtained in a different project (Fig. 4). The Metabolic Process category represented a much larger proportion of the leaf proteome, and this reflects the greater number of leaf proteins associated with photosynthesis (Supplementary Table S1). In contrast, the stigma proteome was noticeably enriched in a number of categories including Cell Wall Modification, Cytoskeleton, Defence, Lipid Metabolic Process, Protein Metabolic Process, Signalling and Transport. These results are only in partial agreement with the functional classification of the transcripts enriched in the mature triticale stigma (Tran *et al.*, 2013), and this may be due to the fact that the gene expression enrichment analysis was much more comprehensive and compared all vegetative and reproductive triticale tissues. Considerably more proteomic data from Triticeae tissues will be required for a thorough comparison.

### Stigma proteins with a signal peptide

Many of the interactions between the stigma and the pollen grain/tube are extracellular and, although not all extracellular proteins possess signal peptides (Drakakaki and Dandekar, 2013), the presence of a signal peptide is a reliable indicator of protein secretion (Lum and Min, 2011) and may point to stigma proteins involved in extracellular interactions. We identified 158 proteins predicted to contain a signal peptide representing 7.9% of the total number of stigma proteins with a start codon (Supplementary Table S1). As the proportion of all the triticale proteins with a signal peptide is unknown, it is impossible to determine whether this percentage represents a possible enrichment in the stigma. However, as a comparison, about 14% of all the maize proteins are predicted to have a signal peptide (Xu *et al.*, 2012), so it seems possible that the low proportion of secreted proteins identified in this study represent specialized proteins that need to be present for the initial interactions with the pollen whereas additional proteins are generated as the pollen tube grows through the stigma. The great majority of the triticale stigma proteins with a signal peptide were found within only five functional categories: Protein Metabolic Process (36%), Carbohydrate Metabolic Process (21%), Cell Wall Modification (18%), Defence (10%), and Transport (8%) (Supplementary Fig. S2 at *JXB* online). The Protein Metabolic Process category consisted primarily of proteins such as heat-shock proteins/luminal-binding proteins and protein disulfide isomerases, which are probably important in maintaining protein integrity (Supplementary Table S1). It also included several protease inhibitors and proteases and, although proteolytic enzymes can have significant roles in reproduction such as defence, signalling, and programmed cell death (Radlowski, 2005), their potential function in the triticale stigma remains unknown. Many of the proteins in the Carbohydrate Metabolic Process class were also found in the Cell Wall Modification category, and the need for their extracellular localization is in line with a role in allowing and supporting pollen-tube growth. The proportion of extracellular proteins in the Defence category was higher than the value for the total stigma proteome (Fig. 3), again indicating a requirement for these proteins at the site of pollen-tube penetration. The Transport category included more than half of the lipid transfer proteins and this could be due for example to requirements for cuticle synthesis and maintenance.

### Correlation between triticale mature stigma mRNA and protein expression

**Fig. 5.**
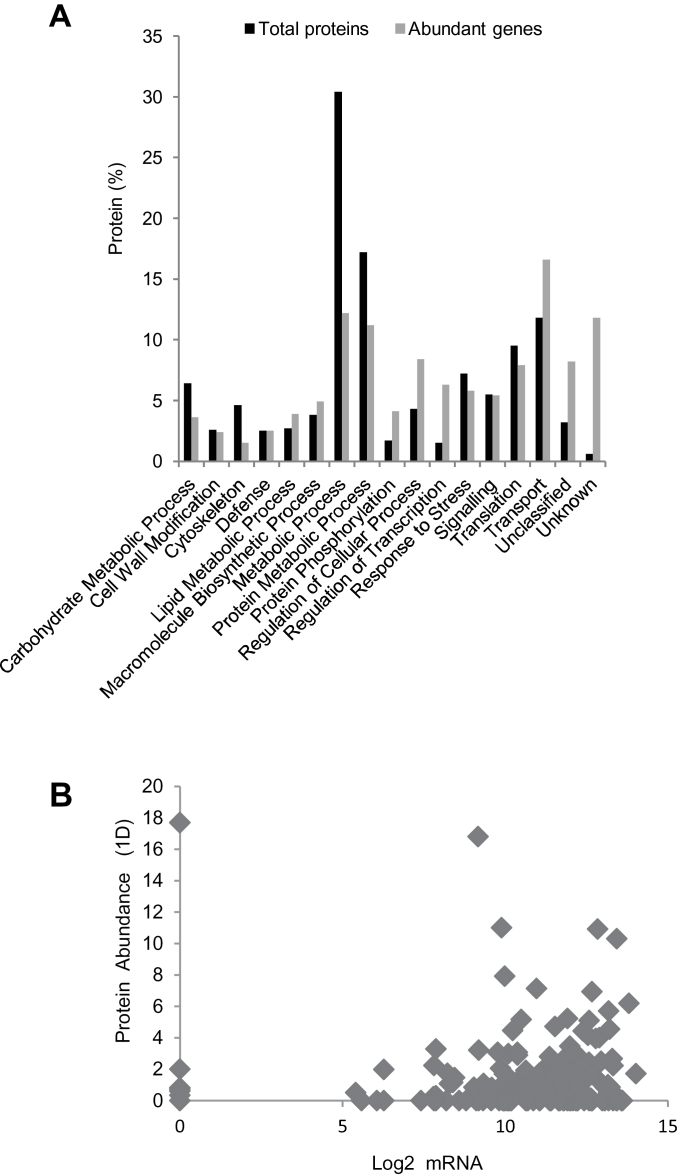
(A) Comparison of the functional distribution of the 2184 proteins and most abundantly expressed genes in the triticale mature stigma. (B) Comparison of triticale mature stigma protein and mRNA abundance. Pearson correlation coefficient, *R* ^2^ = 0.267.

Based on the assumption that the 2184 proteins identified in the current study are biased towards the most abundant proteins (Feng *et al.*, 2009), their functional distribution was compared with that of the 2184 most abundantly expressed stigma genes identified in a microarray analysis (Tran *et al.*, 2013) and the results are shown in Fig. 5A. Of the 2184 genes, 238 showed enriched expression in the pistil when compared with all other triticale tissues, and 179 of those genes showed enriched expression in the stigma (Supplementary Table S1). While similarities were observed in the number of expressed genes and proteins found in certain functional categories, differences were also evident; for example, the proportion of proteins associated with the categories Cytoskeleton and Metabolic Process was considerably higher than that of the associated genes, whereas the reverse trend was observed for Protein Phosphorylation, Regulation of Transcription, Regulation of Cellular Process and Transport (Fig. 5A). These observations are in general agreement with reports describing metabolic and structural genes as being stable and having a high protein/mRNA ratio, unlike proteins involved in regulatory processes, which may require more rapid production and turnover in reaction to a stimulus (Vogel and Marcotte, 2012). Given that the mature stigma has essentially stopped growing and stands in readiness for pollination, it is possible that it accumulates mRNA encoding regulatory and transport genes to be rapidly translated upon pollen adhesion, hydration, germination, and tube growth.

In order to explore further the relationship between production and degradation of individual mRNAs and proteins, 180 proteins from the 1D LC-MS/MS analysis that were found to specifically match genes present in the microarray analysis (Tran *et al.*, 2013) were used to compare corresponding mRNA and protein levels (Supplementary Table S1). There was very little correlation between the level of mRNA and that of the corresponding proteins (*R* ^2^=0.267) within this relatively small dataset (Fig. 5B). Such a low correlation has also been described for the *Arabidopsis* floral proteome (Feng *et al.*, 2009) and not only emphasizes the importance of both post-transcriptional and post-translational processes but also confirms the importance of investigating both the transcriptome and the proteome.

## Conclusion

To our knowledge, this is first investigation of the Triticeae stigma global proteome. Numerous proteins likely to play significant roles in stigma development, function, and protection of the next generation were revealed and should provide the basis for future research aimed at a better understanding of cereal pollen–stigma interactions.

## Supplementary data

Supplementary data are available at *JXB* online.

Supplementary Fig. S1. 1D SDS-PAGE of triticale mature stigma proteins indicating bands excised for 1D LC-MS/MS analysis.

Supplementary Fig. S2. Functional distribution of triticale mature stigma proteins with a predicted signal peptide.

Supplementary Table S1. Triticale mature stigma proteins identified by 1D LC-MS/MS, 2D LC-MS/MS, and OGE LC-MS/MS. List of top 2184 genes expressed in the triticale mature stigma. List of proteins used in the protein/mRNA quantitative comparison. List of wheat leaf proteins.

Supplementary Data

## Abbreviations:

1D: one-dimensional
2D: two-dimensional
DTT: dithiothreitol
IEF: isoelectric focusing
LC-MS/MS: liquid chromatography/tandem mass spectrometry
MS: mass spectrometry
OGE: OFFGEL electrophoresis
RP-HPLC: reversed-phase high-pressure liquid chromatography
TF: transcription factor.
