# Supplementary material for: Proteomic profiling reveals insights into Triticeae stigma development and function
Source: J Exp Bot. 2014 Aug 28;65(20):6069–80. doi: 10.1093/jxb/eru350 (PMC4203142; doi:10.1093/jxb/eru350)
Supplement: Supplementary Data [file supp_65_20_6069__index.html]

Proteomic profiling reveals insights into Triticeae stigma development and function — Proteomic profiling reveals insights into Triticeae stigma development and function — Supplementary Data 

# Proteomic profiling reveals insights into Triticeae stigma development and function

## Supplementary Data

Data files

**Files in this Data Supplement:**

- Supplementary Data - Supplementary Data
- Supplementary Data - Supplementary Data
