# Supplementary material for: Proteomic profiling reveals insights into Triticeae stigma development and function
Source: J Exp Bot. 2014 Aug 28;65(20):6069–80. doi: 10.1093/jxb/eru350 (PMC4203142; doi:10.1093/jxb/eru350)
Supplement: Supplementary Data [file supp_eru350_jexbot130526_file002.pdf]

Proteomic profiling reveals insights into Triticeae stigma development and function

Nazila Nazemof, Philippe Couroux, Christof Rampitsch, Tim Xing, and Laurian S Robert

Supplementary Data

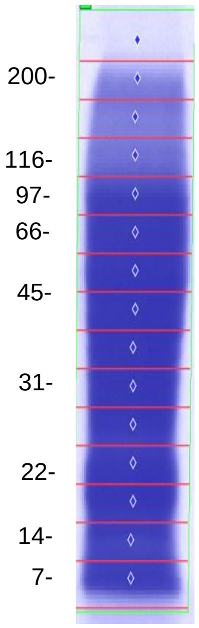

Figure S1. 1D SDS-PAGE of triticale mature stigma proteins indicating bands excised for 1D LC-MS/MS analysis.

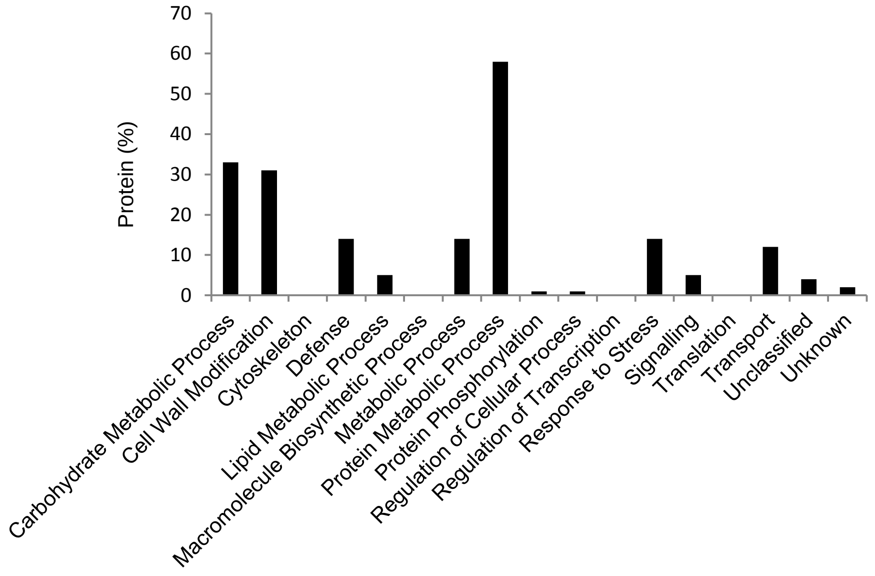

Figure S2. Functional distribution of triticale mature stigma proteins with a predicted signal peptide.
